# The Cost of Loneliness: Ageism and the Politics of Assisted Dying in Chie Hayakawa’s Plan 75

**DOI:** 10.1093/geroni/igaf122.3354

**Published:** 2025-12-31

**Authors:** Ulla Kriebernegg

**Affiliations:** University of Graz, Graz, Steiermark, Austria

## Abstract

This paper examines ageism and medically assisted dying as depicted in the Japanese dystopian film Plan 75 (2022), which envisions large-scale, state-organized gerontocide as a solution to the so-called “problem” of old age. In the film, healthy individuals aged 75 and above who voluntarily enroll in the program receive a financial incentive, enabling them to partake in final experiences such as a domestic journey or a luxurious meal before they are put to death in a clinic. The narrative disturbingly normalizes ageism, presenting voluntary death as a practical response to loneliness, poverty, and depression—while simultaneously alleviating Japan’s economic crisis. Using a cultural gerontological framework, this study employs film analysis to interrogate how Plan 75 constructs narratives about aging, autonomy, and societal worth. The paper examines the depiction of older adults as isolated, burdensome figures whose existence must be negotiated through institutionalized solutions rather than through meaningful social interventions and compassionate care. The study also considers how Plan 75 reflects broader social inequalities, particularly in relation to class, gender, and government control over aging populations. Finally, this paper highlights the role of cinema in shaping public perceptions of aging, influencing policy debates on end-of-life care, and challenging ageist narratives in both medical and social contexts.

